# Rab46: a novel player in mast cell function

**DOI:** 10.1093/discim/kyad028

**Published:** 2023-12-22

**Authors:** Lucia Pedicini, Jessica Smith, Sinisa Savic, Lynn McKeown

**Affiliations:** Leeds Institute of Cardiovascular and Metabolic Medicine, Faculty of Medicine and Health, University of Leeds, Leeds LS2 9JT, UK; Leeds Institute of Cardiovascular and Metabolic Medicine, Faculty of Medicine and Health, University of Leeds, Leeds LS2 9JT, UK; Department of Clinical Immunology and Allergy, St James’s University Hospital, Leeds, UK; National Institute for Health Research-Leeds Biomedical Research Centre and Leeds Institute of Rheumatic and Musculoskeletal Medicine, Wellcome Trust Brenner Building, St James’s University Hospital, Leeds, UK; Leeds Institute of Cardiovascular and Metabolic Medicine, Faculty of Medicine and Health, University of Leeds, Leeds LS2 9JT, UK

**Keywords:** mast cells, EFCAB4B, Rab46, CRACR2A, histamine, degranulation, trafficking, Rab GTPases

## Abstract

Mast cells are infamous for mediating allergic and inflammatory diseases due to their capacity of rapidly releasing a wide range of inflammatory mediators stored in cytoplasmic granules. However, mast cells also have several important physiological roles that involve selective and agonist-specific release of these active mediators. While a filtering mechanism at the plasma membrane could regulate the selective release of some cargo, the plethora of stored cargo and the diversity of mast cell functions suggests the existence of granule subtypes with distinct trafficking pathways. The molecular mechanisms underlying differential trafficking and exocytosis of these granules are not known, neither is it clear how granule trafficking is coupled to the stimulus. In endothelial cells, a Rab GTPase, Rab46, responds to histamine but not thrombin signals, and this regulates the trafficking of a subpopulation of endothelial-specific granules. Here, we sought to explore, for the first time, if Rab46 plays a role in mast cell function. We demonstrate that Rab46 is highly expressed in human and murine mast cells, and Rab46 genetic deletion has an effect on mast cell degranulation that depends on both stimuli and mast cell subtype. This initial insight into the contribution of Rab46 to mast cell function and the understanding of the role of Rab46 in stimuli-dependent trafficking in other cell types necessitates further investigations of Rab46 in mast cell granular trafficking so that novel and specific therapeutic targets for treatment of the diverse pathologies mediated by mast cells can be developed.

## Introduction

**Figure 1:**
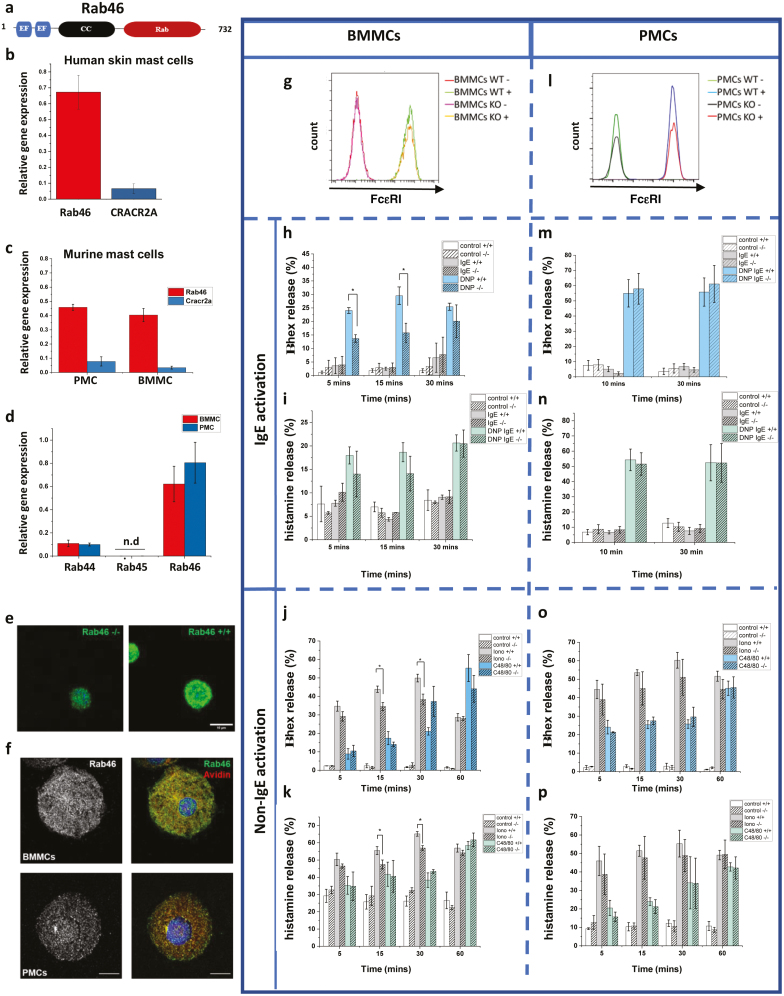
**The role of Rab46 in mast cell degranulation**. (a) Schematic depicting the 732 amino acid length of Rab46, including the EF-hands (Ca^2+^-sensing), coiled-coil, and Rab domains. (b–c) qPCR analysis of relative Rab46 mRNA expression compared to CRACR2A in human mast cells (b) and (c) murine peripheral mast cells (PMCs) and bone marrow mast cells (BMMCs). (d) Relative mRNA expression of other large Rab GTPases (Rab44, Rab45 compared to Rab46) with similar sequence and domain structure to Rab46. (e) Airyscan confocal images of anti-CRACR2A/Rab46 staining (green) in wild type (Rab46^+/+^) and Rab46 knockout (Rab46^−/−^) BMMCs. Scale bar = 10 μm. (f) Rab46 (left images: gray) distribution in BMMCs (top) and PMCs (bottom) and localization compared with avidin (merged images: avidin red and Rab46 green). Nuclei = Hoescht (blue). Scale bar = 10 μm. (g) Representative traces from FACs analysis of FcεRI expression in BMMCs extracted from Rab46^+/+^ (WT) and Rab46^−/−^ (KO) mice with and without IgE stimulation (WT+ or WT−/KO+ or KO−). (h–i) Time-dependent measurement of IgE-mediated β-hexosaminidase (h) and histamine release (i) in BMMCs extracted from Rab46^+/+^ and Rab46^−/−^ mice. BMMCs were acutely (1 h) pre-incubated with anti-DNP-IgE (IgE) or control (control) and challenged where indicated with human serum albumin DNP (IgE DNP: blue and green columns). (j–k) Time-dependent measurement of ionomycin and C48/80-mediated β-hexosaminidase (j) and histamine (%) release (k) in BMMCs extracted from Rab46^+/+^ or Rab46^−/−^ mice. (l) Representative traces from FACs analysis of FcεRI expression in PMCs extracted from Rab46^+/+^ (WT) or Rab46^−/−^ (KO) mice with and without IgE stimulation (WT+ or WT−/KO+ or KO−). (m–n) Time-dependent measurement of IgE-mediated β-hexosaminidase (m) and % histamine (n) release in PMCs extracted from Rab46^+/+^ or Rab46^−/−^ mice. (o–p) Time-dependent measurement of ionomycin and c48/80-mediated β-hexosaminidase (o) and histamine (p) release in PMCs extracted from Rab46^+/+^ or Rab46^−/−^ mice. *N*/*n* = 3/9 for all degranulation experiments. *P < 0.05.

Mast cells store a powerful arsenal of pro-inflammatory mediators that, upon release, mediate a variety of physiological functions such as wound healing and the development of acute inflammation [1]. Mast cells are also directly involved in several pathological conditions and are prominent initiators of allergic diseases [2]. Crosslinking of IgE on the primed mast cell surface causes the characteristic symptoms of allergy by the release of histamine, cytokines, leukotrienes, proteases, and heparin from mast cell granules. However, in order to deliver appropriate physiological responses, it is becoming increasingly clear that the selective release of granule cargo is distinctly coupled to the activating signal, the dysregulation of which leads to the pathological conditions associated with aberrant mast cell degranulation [3]. There are several studies exploring the effects of distinct agonists on the differential release of these pro-inflammatory cargo [4]; however, little is known about the molecular mechanisms underlying the trafficking and secretion of the secretory granules (SGs) in which these mediators are stored [5]. An essential step toward deciphering the mechanisms behind exocytosis is the identification of the cellular components that regulate this process. Rab GTPases are master regulators of intracellular trafficking events, and thereby, they regulate immunity and inflammation responses by controlling granule trafficking and secretion in immune cells [6]. Several conventional Rab proteins are reported to be involved in mast cell exocytosis; however, new evidence has recently pointed to the contribution of a new class of large EF-hand containing Rab GTPases in mast cell degranulation and function [7]. Rab46 (CRACR2A-L; *Ensembl* CRACR2A-203; CRACR2A-a) is a large Ca^2+^-sensing GTPase that, in addition to a Rab-domain, contains a coil-coiled domain and two EF-hand domains (Fig. 1a) [8]. Rab46 is necessary for stimuli-coupled trafficking of a sub-population of endothelial-specific vesicles to the microtubule organizing center [9, 10] and for vesicle translocation to the immunological synapse in T-cells [11]. Here, we investigated the role of Rab46 in regulating mast cell degranulation and in establishing the pro-inflammatory environment typical of many immune-related diseases.

Rab46 (732 amino acids) is one of two functional isoforms translated by the EFCAB4B gene. The other isoform, CRACR2A (CRACR2A-S; *Ensembl* CRACR2a-201; CRACR2A-c), is a shorter (395 amino acids), non-Rab isoform of EFCAB4B and is expressed in T-cells where it regulates store-operated calcium entry and T-cell signaling [12]. Analysis of EFCAB4B isoform expression in mast cells suggests Rab46 is expressed in both murine and human mast cells (Fig. 1b,c). Quantitative qPCR data suggest that Rab46 mRNA expression is five times more abundant than CRACR2A in both murine bone-marrow-derived mast cells (BMMCs) and peritoneal-derived mast cells (PMCs) (Fig. 1c). Moreover, and of particular interest because of the emerging role of these EF-hand containing Rab GTPases in mast cell function [7], we found that among the three known large Rab GTPases (Rab44, Rab45, Rab46), Rab46 mRNA is the most abundant. Rab46 mRNA is seven times more abundant than Rab44 in both BMMCs and PMCs, whereas Rab45 mRNA was not detected (Fig. 1d).

In our previous studies, we demonstrated the role of Rab46 in the trafficking of selective endothelial granules and demonstrated the colocalization of Rab46 with granule markers [9]. Using our previously validated anti-CRACR2A/Rab46 antibody (7), we observed the localization of Rab46 to mast cell granules (Fig. 1e: green). AiryScan confocal analysis of both BMMCs and PMCs (Fig. 1f) revealed that endogenous Rab46 (green) localizes to mast cell granules stained with avidin (in red), although these granules display differing distributions and contain different levels of Rab46 between mast cell sub-types. These results suggest a potential role of Rab46 in the regulation of mast cell vesicular trafficking.

To determine the effect of Rab46 depletion (Rab46^-/-^) on mast cell degranulation, we measured β-hexosaminidase and histamine secretion in BMMCs and PMCs extracted from wild-type (Rab46^+/+^) mice and mice where Rab46 had been knocked out (Rab46^-/-^). For IgE-based stimulation, BMMCs and PMCs were acutely (1 h) pre-sensitized with either control IgE (control) or monoclonal anti-dinitrophenyl (DNP) IgE, before being challenged with DNP-BSA (shown as IgE DNP on graphs) to aggregate FcϵRI (Fig. 1h, i, m, n). For non-IgE-based stimulation BMMCs and PMCs were directly stimulated with non-IgE stimuli (ionomycin and C48/80: Fig. 1j, k, o, p). Figures g-k represent data from BMMCs, and figures l-p represent data from PMCs. The IgE receptor, FcεRI, expression was comparable between BMMCs (and PMCs) extracted from Rab46^+/+^ and Rab46^-/-^ mice, suggesting potential differences in secretion between the genotypes are not due to Rab-dependent FcεRI trafficking defects (Fig. 1g, l). Firstly, we measured mast cell secretion upon stimulation of the high-affinity IgE receptor (FCεRI) using IgE-pre-sensitized BMMCs. In mast cells pre-treated with anti-DNP IgE (IgE), β-hexosaminidase secretion was significantly (*P* = < 0.05) reduced in BMMCs derived from Rab46^−/−^ mice (blue versus blue stripe columns) compared to BMMCs extracted from Rab46^+/+^ after DNP (“allergy”) challenge (IgE/DNP: Fig 1h). A similar trend was also observed for histamine secretion (Fig. 1i: compare green columns). In addition to FcϵRI stimulation, mast cell degranulation occurs via activation of Mas-related G protein-coupled receptor X2 (MRGPRX2; mouse ortholog MrgprB2). Here, using the MRGPRX2 specific agonist compound 48/80 (C48/80) (Fig. 1j, k: see blue and green columns), the data suggests Rab46 deletion had no significant effect on β-hexosaminidase or histamine secretion in BMMCs. However, measurement of non-immunological activation of mast cells by the calcium ionophore, ionomycin (iono), demonstrated that Rab46 depletion induces a significant reduction in both β-hexosaminidase and histamine release (Fig.1j, k: see gray versus gray stripe columns). These results suggest a role for Rab46 in IgE (FcεRI)-mediated secretion and ionomycin-mediated degranulation but not the specific MRGPRX2 pathway in BMMCs.

Interestingly, although Rab46 mRNA expression in PMCs is comparable to BMMCs (Fig 1c), there were no significant differences observed in IgE/DNP-mediated β-hexosaminidase or histamine release between mast cells extracted from Rab46^+/+^ and Rab46^-/-^ mice (Fig. 1m,n). In addition, no significant differences were detected in ionomycin stimulated β-hexosaminidase or histamine release between PMCs extracted from Rab46^+/+^ and Rab46^-/-^ mice (Fig. 1o,p).

These data suggest that although PMCs exhibit a stronger and faster degranulation response compared to BMMCs, the response to the stated agonists appears to be independent of Rab46. On the other hand, Rab46 deficiency significantly impairs BMMC degranulation upon FcεRI and ionomycin stimulation, while activation of the MRGPRX2 pathway triggers a Rab46-independent response in both BMMCs and PMCs.

In this study, we demonstrate for the first time the expression of Rab46, a novel EF-hand containing GTPase in mast cells, and our *in vitro* results suggest a role for Rab46 in exocytosis in BMMCs. Interestingly, in endothelial cells, Rab46 is coupled to specific agonists to mediate the selective trafficking of sub-populations of endothelial-specific vesicles [9]. Both endothelial-specific vesicles and mast cell granules are derived from lysosomes, indicating they may share common regulatory mechanisms. Here, the data implies that Rab46 may also play a role in agonist-specific pathways in some populations of mast cells. Furthermore, the data suggests Rab46 may regulate granule release in mast cells dependent on the mast cell subtype, underlying the importance of mast cell heterogeneity. A greater understanding of Rab46-dependent trafficking events will provide opportunities to develop novel therapeutic targets for the treatment of the increasing prevalence of allergic and other inflammatory diseases.

## Materials and methods

### Ethics

Murine work was carried out in accordance with The Animals (Scientific Procedures) Act 1986 (Amended 2012), and all protocols were authorized by the University of Leeds Animal Ethics Committee and Home Office UK (Project License P606320FB to David J Beech). Mice were kept in the University of Leeds animal facility under standard conditions, including a 12-h sleep/wake cycle, with access to water and chow diet ad libitum.

### Mice

Rab46^−/−^ cells are from CRACR2A tm1.1 (KOMP) vlcg (global knockout mice that were created from ES cell clone 15424A-C4, generated by Regeneron Pharmaceuticals, Inc. and obtained from the KOMP Repository) (www.komp.org). Rab46^+/+^ wild-type cells are derived from C57BL/6N black controls. Genotypes of the mice were validated using real-time PCR with specific probes designed for each gene (Transnetyx, Cordova, TN). Western blots were performed from mast cell lysates to confirm the knockout of the Rab46 protein.

### Cell culture

Bone marrow cells (BMMCs) and peritoneal cells (PMCs) were obtained from each mouse. BMMCs from 8-week-old female mice were cultured at 37°C and 5% CO_2_ in RPMI 1640 medium containing 10% fetal bovine serum, 2 mM L-glutamine, non-essential amino acids, 1% penicillin and streptomycin, and supplemented with 20 ng/ml interleukin-3 and 20 ng/ml Stem Cell Factor (both from Peprotech, Rocky Hill, NJ). After 4 weeks in culture, more than 95% of the cells were mast cells as assessed by FcεRI and c-kit expression by flow cytometry.

Peritoneal cells were obtained by injecting the mice with 5 ml of PBS for the peritoneal lavage. Cells were centrifuged at ~300 × *g* and resuspended in RPMI Medium supplemented with 10% fetal calf serum, 1% PenStrep, non-essential amino acids, 20 ng/ml IL-3, and 20 ng/ml Stem Cell Factor (SCF). Cells were further cultured in 5% CO_2_ at 37°C. On the second day of cultivation, all non-adherent cells were collected and transferred into a new flask. PMCs were used for experiments between 10 and 25 days of culturing. Flow cytometry analysis identified 98.5–99.5% of cells to be double positive for FcεRI and c-Kit and could be ranked as mast cells.

### RT-PCR

RNA isolation was performed using the High Pure RNA isolation kit (Roche) according to the manufacturer’s protocol. Reverse transcription (Applied Biosystems) was followed by real-time PCR using SYBR green using a LightCycler (Roche). Expression levels of housekeeping genes (GAPDH and b-actin) were also measured. Primer sequences are provided in the table below

**Table:** 

mCRACR2A	F: 5ʹ CTGGAGCGACTCAATCAGAAGC 3ʹ R: 5ʹ GAGGCAAGCTGAGTTGGAAGAG 3ʹ
mRab46	**F**: 5ʹ GGGCAGCCTGTTGGAAAAGA 3ʹ **R**: ACTCGGTAGTCGATGCCCAC 3ʹ
mCracr2a + mRab46	**F**: 5ʹ GATGGACAGACTTGGAGCCC 3ʹ **R**: 5ʹ CAGCAATTTTCTTTCTGAGGGCA 3ʹ
mRab44	**F**: 5ʹ GCTGAGCAGACAGTGACCTC 3ʹ **R**: 5ʹ CTGAACCTGGCCTCCTCTC 3ʹ
mRab45	**F**: 5ʹ GGAGATCTGGAGTTACGGTGA 3ʹ **R**: 5ʹ AACACGACTAAGCAGCCACA 3ʹ
hRab46	**F**: 5ʹ GGTCATCCTTGCCTACG 3ʹ **R**: 5ʹ GCTCGCATGAGATCAAGT 3ʹ

### Immunocytochemistry

A total of 5 × 10^4^ MCs were plated on Cell-Tak (Corning)-coated Ibidi 8-well plates. Cells were fixed with 4% PFA, and secretory granules were stained with avidin-FITC (BioLegend) and rabbit anti-Rab46 (CRACR2A) antibody (ProteinTech) for 1 h at RT. After washing in Phosphate Buffered Saline (PBS), a fluorescently labeled secondary antibody (Alexa Fluor 594 anti-goat IgG - Jackson ImmunoResearch Labs) was applied for 30 min. Cells, washed with PBS, were briefly incubated in Hoechst before being mounted with Ibidi mounting medium.

### High-resolution AiryScan microscopy

High-resolution microscopy was performed using an inverted confocal laser-scanning microscope, Zeiss LS880 with AiryScan system. Images were captured using a 63×/1.4 oil objective and 405-nm diode, Argon/2 (458, 477, 488, and 514 nm), HeNe 543 nm, and HeNe 633 nm lasers. All the images were acquired and processed with Zen software. All imaging was performed at room temperature.

### Image analysis

All images were processed using Fuji (Fuji is ImageJ: https://imagej.net/downloads). The images used to compare anti-Rab46 staining in BMMCs from Rab46^−/−^ versus Rab46^+/+^ mice were equally adjusted according to the histogram of the wild-type mouse.

### Flow cytometry

For each mast cell extraction, PMCs and BMMCs were centrifuged at 300 g for 5 mins and 1 × 10^5^ cells per reaction re-suspended in 200 μl flow cytometry (FACs) buffer (500 ml PBS; 2 % FBS; 1 mM EDTA; 25 mM HEPES; pH 7.4) in a FACS tube. Cells were placed on ice and incubated with 1:100 PE-tagged anti-PE-FCεRIa (BioLegend) or APC-CD117 (BioLegend) or negative control for 20 min. After 3× wash, cells were resuspended in 300 μl FACs buffer, and fluorescent measurements were taken on a CytoFlex 2 laser flow cytometer. Analysis and graphs created using FlowJo software.

### Degranulation assay

PMCs and BMMCs for ELISAs were extracted from 3× Rab46^+/+^ wild type and 3× Rab46^−/−^ mice, and all experimental conditions were performed in triplicates (*n*/*N* = 3/9 per genotype). PMCs and BMMCs were centrifuged at 300 g for 5 min and re-suspended in Tyrodes solution containing in mM: NaCl 135; KCl 5; CaCl_2_ 1.8; MgCl_2_ 1; glucose 5.6; HEPES 20; pH 7.4. The cells were seeded in a U-bottom 96-well plate (5 × 10^5^ cells/well). For sensitization, 1 × 10^6^ MCs were incubated with 1 μg/ml of monoclonal anti-DNP IgE (Sigma) for 1 h at 37°C. Degranulation was induced by incubation of BMMCs in the presence of 100 ng/ml Dinitrophenyl human serum albumin (DNP) (10 ng/ml PMCs) or 1 μg/ml IgE or 10 μM C48/80 or 2 μM ionomycin or buffer controls at 37°C and 5% CO_2_. Cells were centrifuged at 300 g for 5 min. The supernatants were separated, and the cell pellets were lysed in ddH2O during 5 min at room temperature and then frozen at −80°C.

### β-hexosaminidase quantification

The amount of released β-hexosaminidase enzyme was quantified by spectrophotometric analysis of 4-Methylumbelliferyl n-acetyl-β-d-glucosaminide (4-MUG) hydrolysis, as previously described. In short, the cell lysates and supernatants were incubated separately with 5 μM 4-MUG in 100 mM citrate buffer for 1 h at 37°C, and the reaction was stopped by adding 100 mM Na2CO3-NaHCO3 buffer (pH 10.7). The hydrolysis rate of 4-MUG was quantified by fluorometric measurements at 460 nm emission and 355nm excitation using the Synergy H1 spectrophotometer. The percentage of β-hexosaminidase release was calculated as the absorbance ratio of the supernatant to the sum of supernatant and lysate. If not stated otherwise, all chemicals were purchased from Sigma.

### Histamine quantification

Histamine content in the supernatant and the cell lysate was measured by a fluorometric method based on its stable condensation with o-phthalaldehyde (OPT) in an alkaline medium. Briefly, supernatants and intracellular contents were collected and exposed to 2.3 μl OPT (7.5 μM) and 9 μl NaOH (1 mM) for 4 min. After stabilization with 3 μl of HCl (3 mM), fluorescence at 360 nm in a conventional spectrophotometer was quantified. The amount of histamine was calculated according to a standard curve and the % of histamine released was reported (the supernatant as a % of the supernatant and lysate).

### Statistics

All average data are represented by mean ± SEM. Paired *t* test was performed as appropriate when the comparison between two data groups was sought. Statistical significance was considered to exist at *P* < 0.05 (*P < 0.05; **P < 0.01). Where comparisons lack an asterisk, they were not significantly different and/or marked as not significant. OriginPro 2017 software was used for data analysis and presentation.

## Supplementary Material

kyad028_suppl_Supplementary_Material

## Data Availability

All data available upon request to the corresponding author

## Abbreviations

BMMC: bone-marrow-derived mast cells
OPT: o-phthalaldehyde
PMC: peritoneal-derived mast cells
SG: secretory granules
