# Supplementary material for: Rab46: a novel player in mast cell function
Source: Discov Immunol. 2023 Dec 22;3(1):kyad028. doi: 10.1093/discim/kyad028 (PMC10917158; doi:10.1093/discim/kyad028)
Supplement: kyad028_suppl_Supplementary_Material [file kyad028_suppl_Supplementary_Material.pptx]

## Slide 1
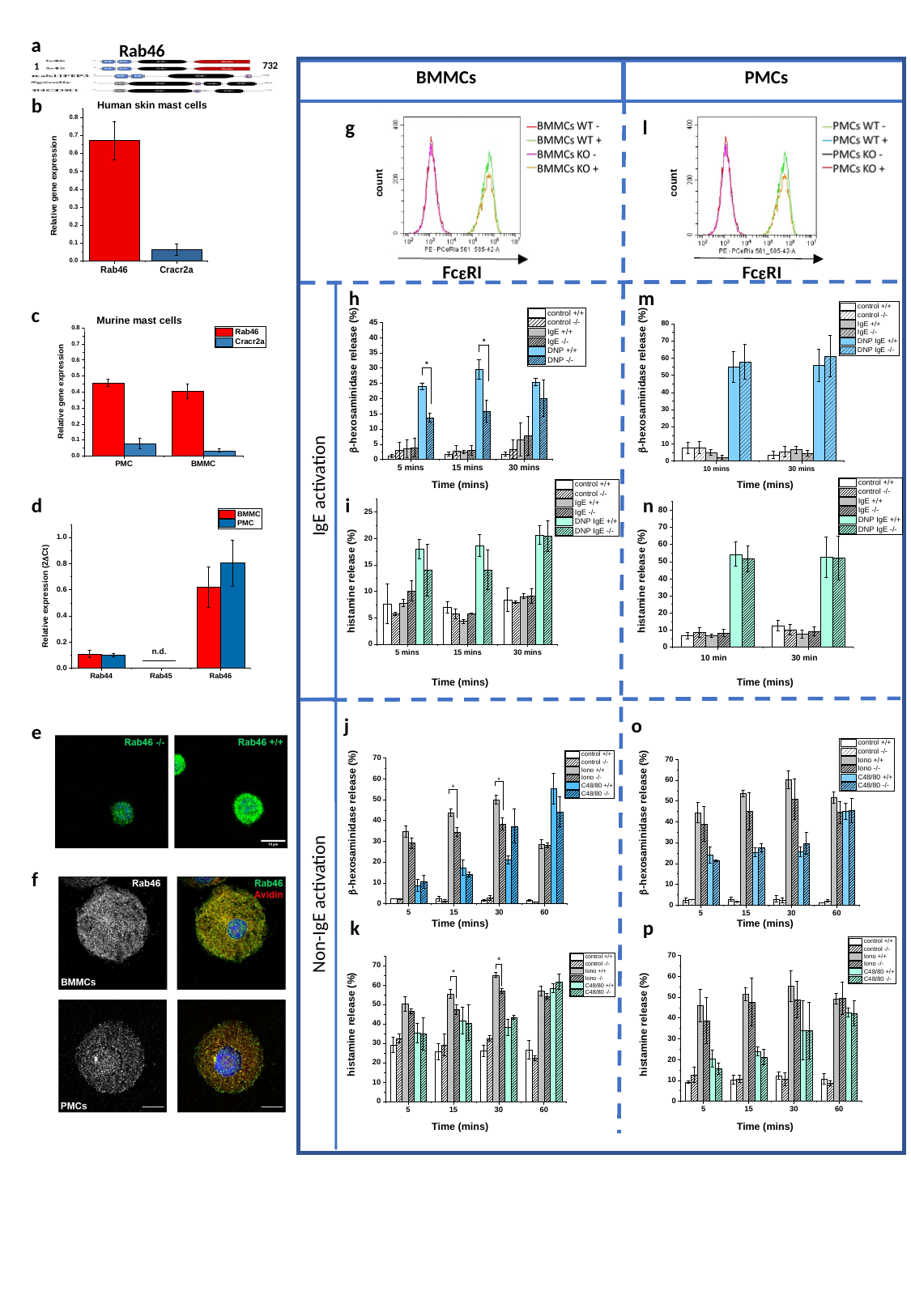

a
Rab46
1
732
BMMCs
PMCs
FceRI
g
l
count
count
FceRI
h
m
b-hexosaminidase release (%)
b-hexosaminidase release (%)
IgE activation
Time (mins)
Time (mins)
i
n
Time (mins)
Time (mins)
Time
j
o
b-hexosaminidase release (%)
b-hexosaminidase release (%)
Non-IgE activation
k
p
Time (mins)
Time (mins)
Time (mins)
Time (mins)
Human skin mast cells
b
c
Murine mast cells
d
histamine release (%)
histamine release (%)
e
f
histamine release (%)
histamine release (%)
